# Fetal age assessment for Holstein cattle

**DOI:** 10.1371/journal.pone.0207682

**Published:** 2018-11-19

**Authors:** Camilla Hessel Krog, Jørgen Steen Agerholm, Søren Saxmose Nielsen

**Affiliations:** 1 Section for Animal Welfare and Disease Control, Department of Veterinary and Animal Sciences, Faculty of Health and Medical Sciences, University of Copenhagen, Frederiksberg, Denmark; 2 Section for Veterinary Reproduction and Obstetrics, Department of Veterinary Clinical Sciences, Faculty of Health and Medical Sciences, University of Copenhagen, Frederiksberg, Denmark; University of Illinois, UNITED STATES

## Abstract

Although transport and slaughter of cattle during the last 10% of the gestation period is prohibited in the European Union, such cattle are sometimes sent for slaughter. The late term pregnancy is usually not recognized by the authorities until the uterus is inspected after slaughter and a near term fetus is observed. Accurate post mortem determination of age of bovine fetuses is therefore of major importance as evidence for the subsequent prosecution of the owner. Fetometric measurements such as crown-rump length (CRL) have been used, but these existing estimators have often been established based on insufficiently described study populations or phenotypes that may have changed in the past decades. Morphological characteristics are also used, but few data are available on the correlation between fetal age and the development of these characteristics. The objectives of this study were to investigate the correlation between fetal age and morphological features of bovine Holstein fetuses and to evaluate the use of these features alone and in combination with fetometric measurements to predict fetal age. We collected fetuses from 274 pregnant Holstein cows with recorded insemination dates slaughtered at a Danish abattoir. Gender, teeth development, occurrence of pigmentation, coat, tactile hair and other morphological features were recorded along with CRL, head width, head length and body weight (BW). The gestational length was calculated based on recorded insemination and slaughter dates, and coefficients of variation (R^2^) were determined for all recorded variables. Notably, the highest R^2^ was recorded for head length (0.985) followed by CRL (0.979) and head width (0.974). The categorical (morphological) variables were less informative. When used in multivariable models, they did offer statistically significance, but for practical purposes, limited additional information. A multivariable model including the fetometric variables head length and width in combination with CRL resulted in R^2^ = 0.99 with predictions that were roughly within +/- 11–12 days in 95% of cases. We conclude that the model based on the fetometric variables only provided the most precise predictions, while combination with morphological features such as eruption of teeth, pigmentation and coat mostly increased the width of the prediction intervals.

## Introduction

Transport and slaughter of pregnant cattle in the last 10% of the gestation period is prohibited in the European Union [1] but Germany recently implemented more strict regulations, e.g. banning slaughter of pregnant cattle during the last 33% of gestation [2]. The authorities enforcing these regulations are however in many cases challenged because mating data is often non-existing or non-accessible. Also, the presence of late term pregnancy in cattle admitted to slaughter is mostly not recognized by the authorities until the uterus is visually inspected after slaughter and the determination of fetal age is therefore usually based on fetometric measurements such as body weight (BW), crown-rump length (CRL) and certain morphological characteristics, e.g. length of coat, size of placentomes and tooth eruption.

When prosecuting herd owners who are accused of having sent late term pregnant cattle to slaughter, declarations from veterinary surgeons are considered significant evidence. It is therefore of outmost importance that such declarations are solid and evidence based. Scientific data for age determination of bovine fetuses, and especially late term fetuses, is however sparse. Assessment of fetal age based on morphological findings such as presence of coat and tactile hair was reviewed by Evans and Sack [3] based on studies published from 1909 to 1965. However, the studies were based only on a few animals and mostly not defined in details. It is therefore difficult to apply these data in forensic cases, also because age variation for each trait has not been provided. Information on morphological and fetometric data of purebred Jersey fetuses and neonates collected until 1983 were provided by Richardson et al. [4], whereas Rexroad et al. [5] provided an estimator of CRL based on 229 purebred Holstein-Friesian fetuses sampled between 1950 and 1971. In both instances, information on underlying data was very sparse and the quality of the reports insufficient according to current standards [6]. Reliable data exist for fetuses in early pregnancy and have been achieved mainly by ultrasonography with the aim to enable accurate age determination in breeding management [7]. Fetometric measurements such as crown-nose length, biparietal braincase diameter, thoracic diameter and growth rates for limbs and various organs [8] have enhanced accuracy but are limited to the first six months of gestation and therefore not of value in relation to violation of the transport and slaughter ban on late term pregnant cattle.

Because of the legislation on transport and slaughter of cattle in the last 10^th^ of gestation in the European Union and poor quality of existing data on late term fetuses, we performed a study to provide solid data for age estimation of bovine fetuses. If a gestation length of 280 days is considered, then the last 10^th^ would correspond to day 253 and onwards. But as fetometric measurements such as BW and CRL are breed dependent, especially during late gestation where the fetus grows rapidly, each cattle breed or at least comparable breeds must be analyzed separately. In Denmark as in other major dairy producing countries, the Holstein breed is a predominant breed and as most Holstein cows are inseminated and data registered in a central data base, reliable breeding data exist for most animals. We therefore performed a study on Holstein fetuses to: a) estimate gestational age at development of certain morphological features such as fur coat, pigmentation and tooth eruption; b) develop a tool to estimate the age of Holstein fetuses based on morphological features along with head length, head width, CRL and BW, c) and apply this tool to identify features of relevance to record across gestation.

## Materials and methods

### Study design and setting

The study was carried out as an observational cross-sectional study at a major Danish cattle abattoir (Danish Crown Beef, Aalborg, Denmark). Approximately 250,000 cattle > 2 years of age are slaughtered in Denmark annually of which 20% are slaughtered at this specific abattoir (2012–2014 data from the national meat inspection). In the present study, data were collected from 20^th^ of March 2017 to 31^st^ of March 2017 and from 18^th^ of April 2017 to 27^th^ of April 2017. The cows were only examined after slaughter. Although multiple legislative texts use relative reference to the gestation length to standardize across species and breeds, the standard gestation length differs between breeds and among animals within each breed, e.g. heifers vs. cows. Here, the mean pregnancy length of 281 days for Danish Holstein cows in 2014 was used as an absolute measure of gestation length, if required.

### Cows

The selection of cows included two steps: First, one of the authors (CHK) examined the uterus for pregnancy through direct visual inspection and palpation at the processing line. Pregnant uteri were opened within 45 minutes of exsanguination. The fetus was sampled and further examined within two hours. A unique dam identity was labeled to each fetus. Second, information about the dams’ breed and most recent insemination date was obtained from the Danish Cattle Database (SEGES P/S, Aarhus, Denmark). Dams not recorded as Holsteins, dams with no artificial insemination date recorded in the past year, dams with twins and dams not recorded as slaughtered on the specific date of recording were excluded.

### Variables used

**Fig 1:**
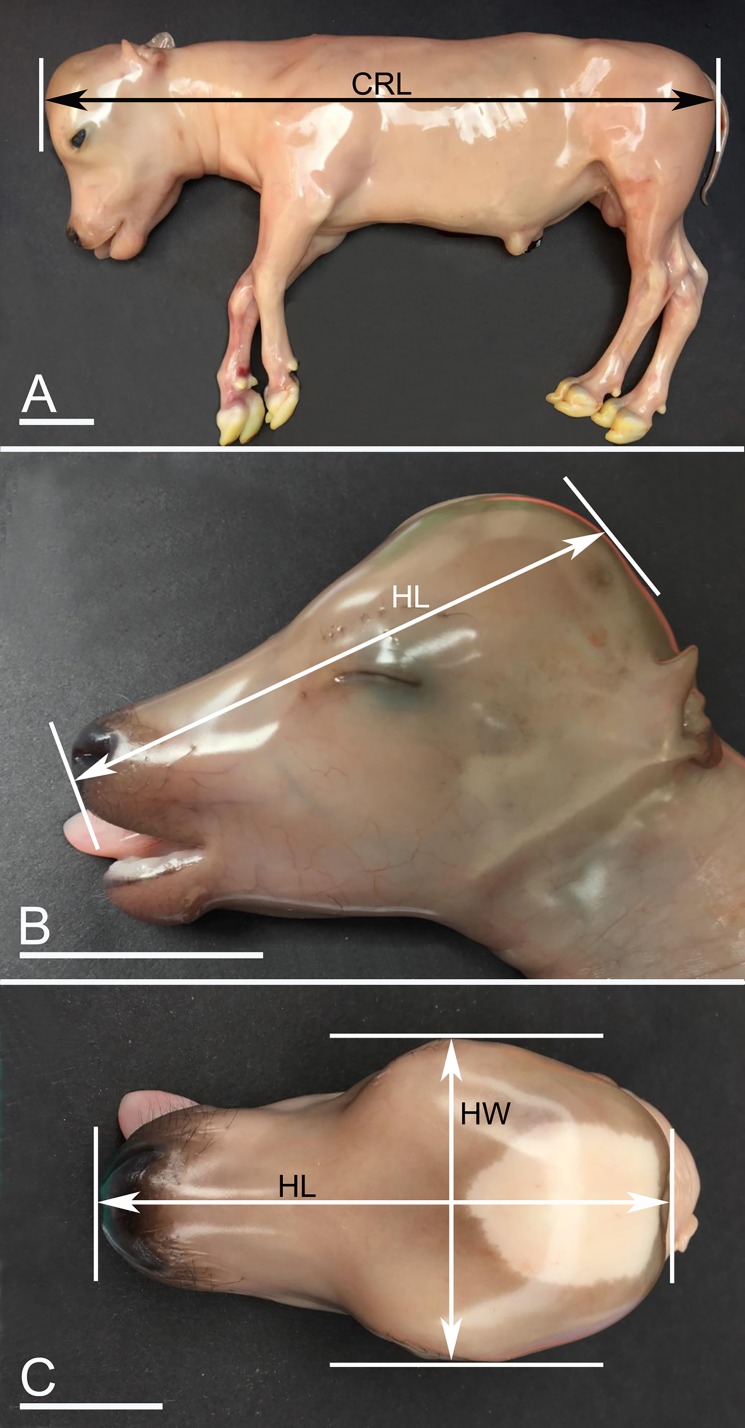
Fetometric measurements. A) Crown-rump length (CRL). This is measured from the most anterior point of the calvarium with the neck flexed 90° to the spine until the most caudal part of the thigh (tuber ischiadicum), fetus aged 145 days. B) Measurement of the head length (HL) from philtrum nasi to the most caudal point of the head. The measurement is done in the dorsal longitudinal midline as indicated in C). The most caudal point varies among fetuses. In younger fetuses, it is more ventral than in late term fetuses, where the intercornual protuberance becomes the most caudal point. Fetus aged 134 days. C) Measurement of the head width (HW) as the largest distance between the zygomatic arches. The site of measuring HL is also indicated. Same fetus as in B). A-C: bar = 5 cm.

The outcome variable was gestation length, which was calculated in days being the insemination date subtracted from the date of slaughter. The predictor variables are listed in Table 1. Fetometric measuring is shown in Fig 1, whereas certain morphometric characteristics are shown in S1 Fig. All recordings were done by CHK, who was blinded to the insemination date and breed of the dam. Raw data are provided in the S1 Dataset.

**Table 1:** Fetal variables recorded at post mortem examination of Holstein fetuses.

Variable	Levels and explanation
Gender	Male; female; external genitalia not yet differentiated.
Genital tubercle	Without; male with a scrotum; female with a clitoris and mammary anlage.
Testicles	Not descended; unilateral; bilateral; not applicable.
Tongue papillae	Absent; Large back (conical); Large front (fungiform); Completely developed.
Eyelids*	Non-separated eyelids covering bulbus oculi recorded as Present; otherwise Absent.
Eyelids in larger fetuses*	Open (eyelids completely opened bilaterally); Closed (when eyelids fused); Partially open (otherwise).
Tactile hair	Recorded for each of the areas: a) Muzzle; b) Periocular; c) Eyelashes. Each recorded with three levels: i) None; ii) Hair follicle present; iii) Hair visible.
Coat	Recorded for each of the locations: a) Base of ear (at transition from external meatus to auricle); b) Inside ear (entire ventral part of external auditory meatus); c) Eyelid (only non-tactile hair); d) Tail (from anal fold to tip of tail); e) Horn bud; f) Coronary band front limbs (any hair above the interdigital cleft to coronary band and pastern); g) Coronary band hind limbs; h) Carpus (entire pastern covered in hair and hair extended dorsally); i) Tarsus (entire pastern covered in hair and extended to the plantar region above the calcaneus); j) Dorsum; k) Hair on the lateral aspects of thorax and abdomen; l) Area around tuber ischiadicum extending distally to the popliteal region, and the perineum); m) Fully (hair all over).
Pigmentation	Pigmentation was recorded for the areas: a) Muzzle; b) Eyelids; c) Lips; d) Ears; e) Limbs; f) Neck; g) Tail; h) Back; j) Full body; and was recorded as i) Present or ii) Absent based on visual inspection. Darker areas were primarily used.
Head length	From philtrum nasi to the most caudal point of the head when measured in the dorsal longitudinal midline (Fig 1). Measured with digital caliper with a precision of 0.01 mm.
Head width	The farthest distance between the zygomatic arches (Fig 1). Measured with digital calipers with a precision of 0.01 mm.
Body weight (BW)	Fetuses with a body weight below 3 kg were measured with Funktion scales with a precision of 1 g. Larger fetuses were measured with an OBH scale with a precision of 0.5 kg. Any remaining umbilical cord was removed at the epidermal junction of the umbilical cord and the fetus prior to weighing.
Crown-rump-length (CRL)	CRL was measured from the crown of the forehead to the caudal border of tuber ischiadicum. The fetus was placed in lateral recumbency with a flexed neck and forehead perpendicular to the dorsal line of the spine. The upper hind limb was placed cranially to the lower hind limb to prevent pelvic rotation. Fetuses too young to be placed as described were measured from the cephalic flexure to the base of the tail.
Deciduous teeth	The incisor teeth and the dens caninum were inspected bilaterally and recorded as: i) Absent (Anlage not visible on the gingival surface); ii) Present but not erupted; iii) Present and erupted. A metal object (blade of a knife) was used to discriminate ii) from iii) in case of doubt. While striking the upper margin of the tooth with the blade, either a muffled sound or a hard sound was created indicating ii) or iii), respectively.

*) There are no eyelids initially but once formed, they fuse to later separate.

### Bias

Blinding of the recorder was done to avoid recording bias, and use of a single recorder was done to reduce information bias. The individual variables (Table 1) are likely to confound each other in a multivariable model, while the developmental stages are overlapping.

### Sample size

The sample size was arbitrarily selected to fit the time-frame of four weeks of sampling with one recorder. No sample size calculation was performed due to the high number of variables and limited prior knowledge of the uncertainty of most variables, where assumptions would have been numerous and benefits limited.

### Statistical analyses

The fetometric measurements, i.e. the quantitative variables BW, head width, head length, CRL, and gestational age at slaughter (GAS), were kept on the recorded units. “Gender” was dichotomized into “differentiated” and “non-differentiated” following a t-test showing no difference in GAS between male and female fetuses. Otherwise, the variables were kept as described in Table 1.

The GAS was described for each stratum of the categorical variables based on the minimum, the 2.5-, 5-, 50-, 95- and 97.5-percentiles, and the maximum. These values were subsequently used to create an interval with 95% of the observations for each group: for dichotomous variables, the lower interval consisted of the minimum to the 95- percentile, and the upper interval consisted of the top 5%. This was also done for variables with more than two categories, but for middle categories, the middle 95% of the observations were used. This procedure was done in an attempt to exclude observations that were possibly a result of erroneous recording of the insemination resulting in pregnancy. Scatterplots where produced for the quantitative variables, and polynomials were included if a visual inspection suggested that a non-linear effect was present, combined with the theoretical consideration that BW is expected to increase exponentially, whereas the others are expected to increase linearly.

Subsequently, the association between GAS (as outcome) and each of the variables in Table 1 as individual predictors was determined using analysis of variance (using the lm()-function in R, Vienna, Austria), and the coefficients of determination were used for numerical comparison of the variables. Finally, multivariable models were constructed as examples to assess possible improvements of the coefficient of determination for specific ages. Two examples were used i.e. one around last third of gestation and one for the last 10^th^ of gestation. These two examples were selected to demonstrate any gain in a multivariable model over a univariable model for two time-periods identified of potential (if pregnancy is in last third of gestation or not [9] or if pregnancy is in last 10^th^ of gestation or not [1]). The models were thus constructed with: GAS as the outcome and all quantitative variables as potential predictors. Furthermore, information on dental variables were included for the model on the last 10^th^ of gestation, and coat variables along with information on whether eye lids were open or not were assessed for the other model. These variables were selected based on the results according to the procedure above. The models were reduced using the Akaike Information Criterion, and the assumptions of normally distributed residuals and variance heterogeneity was assessed for the final model.

The resulting model was validated on a new dataset consisting of additional 71 fetuses from 71 Holstein cows at the same abattoir using similar methods as described above, and the predictive capability was compared to the Rexroad-estimator: GAS = 8.4 + 0.087 x CLR + 5.46 x CRL^0.5^, where CRL is the crown-rump length in mm [5].

## Results

Fetuses were obtained from 294 Holstein cattle and 130 cattle of other breeds, which were subsequently excluded. Of the Holsteins, one had twins, 18 had no recorded insemination date and one had an erroneous date of slaughter. These were also excluded with a resulting inclusion of 274 fetuses from 274 Holstein cattle, with parities as: Parity 0: 8 heifers, parity 1: 47 cows, parity 2: 83 cows, parity >2: 136 cows (S1 Dataset includes all data on the 274 observations).

**Fig 2:**
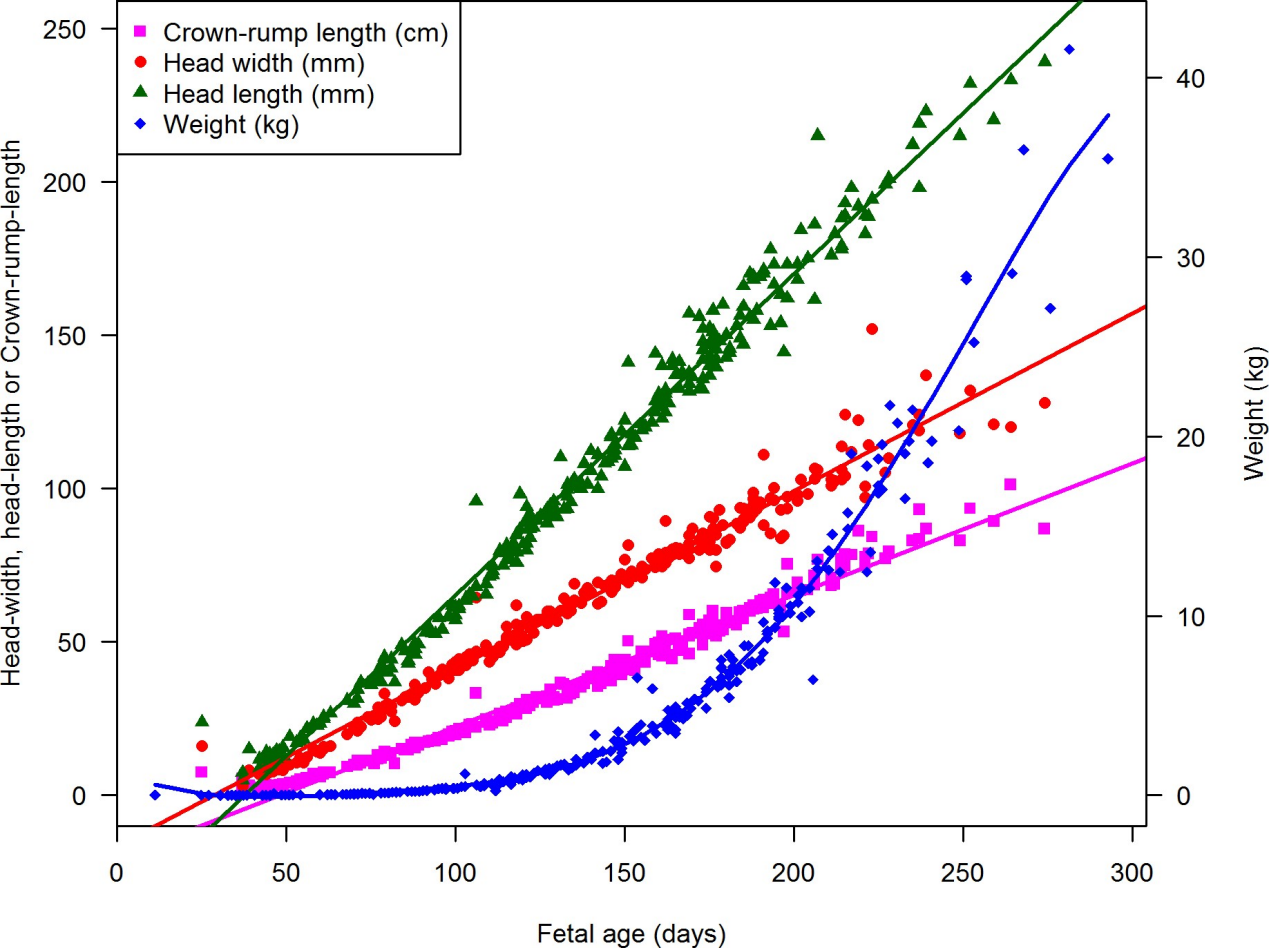
Relation between fetal age, crown-rump length, head width and head length. Scatter plots illustrating the distributions and relations of the fetal age with crown-rump length (CRL) in cm (pink), head width in cm (red), head length in cm (green and body weight (BW) (blue) The points illustrate the observations, while the lines are illustrations of linear models fitted CRL, head-width and head-length, whereas a 5-degree polynomial model was fitted the BW. Notice that the Y-axis values are either in mm or cm depending on the parameter analyzed.

**Fig 3:**
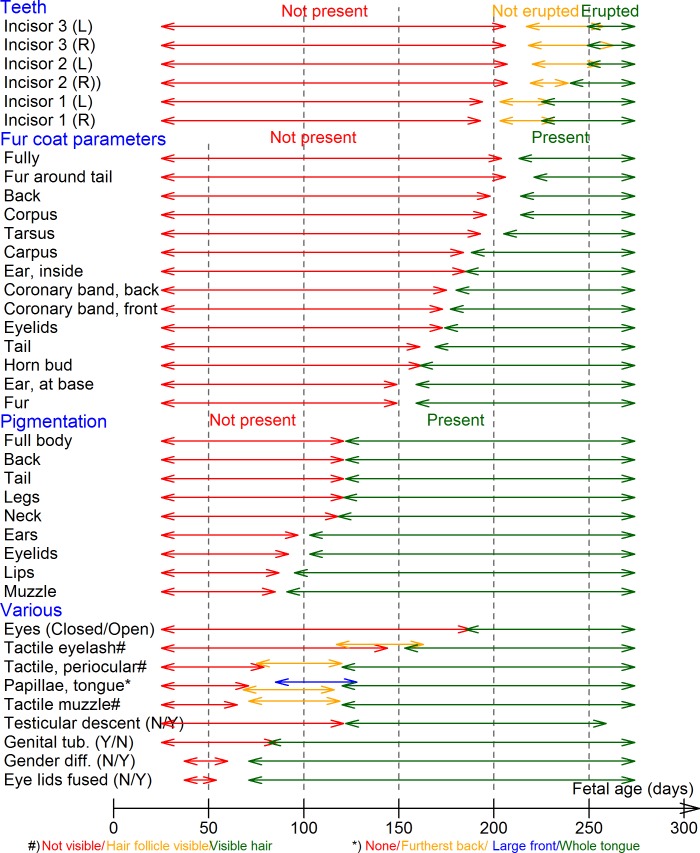
Sequence in the development of fetal morphological characteristics. Fetal morphological characteristics illustrated by the range of 95% of the observations within each group of categorical variables for 247 Holstein fetuses. For the lower (left) group, the 95% is from minimum to the 95-percentile. For the upper (right) group, the 95% cover the 5^th^ percentile to maximum, and for the remaining groups covers the 2.5- to 97.5-percentile range.

The quantitative variables are given in Fig 2 as a function of the GAS, suggesting linear relationships for CRL, head width and head length, and a polynomial 5^th^ order relationship for BW. The number of observations for each stratum of each categorical variable, and the minimum, maximum and 2.5, 5, 50, 95 and 97.5 percentiles are given in S1 Table. The 2.5 and 97.5 percentiles for each stratum of each variable were then used to illustrate fetal morphological developmental characteristics (Fig 3). Notably, gender differentiation and fused eyelids (there are initially no eyelids, but once formed they fuse for later to separate) were the first features to be present, whereas first eruption of an incisor tooth occurred after 200 days of gestation and was among the latest features to develop. Pigmentation developed from around 80–120 days of gestation, whereas presence of coat began from 160 days and progressed onwards over the entire body surface. Thus, the coat variables were chosen as an example of multivariable prediction of transition from gestation month 6 to 7 and eruption of incisor teeth and eyelid-opening used for multivariable prediction of the transition from gestation day 256 into the last 10^th^ of the gestation, both in combination with all of the quantitative variables. The latter were included as linear terms, except for BW, which was included with linear, quadratic, cubic, 4^th^ and 5^th^ order terms (as suggested in Fig 2), but reduced based on the Akaike Information Criterion to have as good a fit as possible. Gestation day 256 is critical regarding violation of the ban on transport and slaughter of cattle being pregnant in the last 10^th^ of gestation.

The estimated coefficients of variation (R^2^) are given in Table 2. Head length had the highest R^2^ with 0.985, followed by CRL (0.979), and head width (0.974). BW on the other hand had a much lower R^2^ value (0.885). The R^2^’s of the morphological parameters (categorical variables) were expectedly lower, with occurrence of tactile hair on the eyelashes as the highest (R^2^ = 0.706). Incisor teeth variables were among those with lowest R^2^ from the univariable analyses. Examples of multivariable models are shown in Table 3, addressing the period from the transition from gestation month 6 to 7 based on hair, eyelids and quantitative variables, and the transition to the last 10^th^ of gestation based on eruption of incisor teeth and quantitative variables, respectively. The R^2^’s were increased to 0.990 and 0.989, respectively, suggesting a slightly better predictive capacity. However, the teeth variables were excluded from the multivariable model for the last 10^th^ of gestation, because inclusion of data on teeth eruption did not improve the R^2^ estimate.

**Table 2:** Coefficients of variation (R^2^) for quantitative and morphological variables prediction of fetal age in Holstein cattle based on examination of 274 fetuses.

Type	Category	Variable	R^2^
Quantitative (fetometric)	N/A	Head length	0.985
	N/A	Crown-rump length	0.979
	N/A	Head width	0.974
	N/A	Body weight (polynomial)	0.885
Categorical (morphological)	Hair	Ear, base	0.660
		Coat (any)	0.641
		Tail	0.594
		Hornbuds	0.585
		Eyelids	0.510
		Coronary band, front	0.506
		Coronary band, back	0.495
		Ear, inside	0.408
		Carpus	0.397
		Tarsus	0.307
		Corpus	0.273
		Dorsum	0.255
		Full body	0.210
		Tail base	0.197
	Tactile hair	Eye lash	0.706
		Muzzle	0.698
		Eye brow	0.697
	Pigmentation	Complete	0.646
		Tail	0.640
		Back	0.639
		Limb	0.636
		Neck	0.629
		Eyelid	0.567
		Ear	0.565
		Lips (upper and lower)	0.510
		Muzzle	0.478
	Teeth	Incisor 1 (Right)	0.316
		Incisor 1 (Left)	0.308
		Incisor 3 (Left)	0.197
		Incisor 3 (Right)	0.196
		Incisor 2 (Right)	0.175
		Incisor 2 (Left)	0.174
	Various	Tongue papillae	0.712
		Testicular descent	0.553
		Genital tubercle	0.428
		Eyelids opened	0.362
		Gender differentiation	0.263
		Eyelids present	0.239

**Table 3:** Multivariable models for coat, eyelid opening and quantitative variables (Model 1, left) and teeth variables and quantitative variables (Model 2, right) for prediction of fetal age (days) in 274 Holstein fetuses.

		Model 1		Model 2	
Variable	Level	Estimate	Std. error	Estimate	Std. error
Intercept		37.763	1.129	32.981	1.751
Head length (mm)		0.445	0.060	0.422	0.058
Head width (mm)		0.343	0.077	0.359	0.074
Crown-rump length (cm)		0.907	0.170	0.984	0.166
Body weight (kg)		-0.159	0.823	-73.41	18.67
Weight^2^		-0.190	0.082	34.13	8.62
Weight^3^		0.00871	0.00342	N/A	N/A
Weight^4^		-0.000106	0.000046	N/A	N/A
Hair dorsally on carpus	Present	6.956	1.814	N/A	N/A
Hair, coronary band, front	Present	3.084	1.795	N/A	N/A
Hair, base of tail	Present	6.903	2.760	N/A	N/A
R^2^	0.990			R^2^	0.989

When the model including fetometric measurements (quantitative variables, Table 2) was used for prediction on the 71 fetuses in the validation dataset, the median difference was– 1 day (0^th^ to 4^th^ quartiles: -29, -5, -1, +2 and +13 days), whereas the Rexroad-estimator resulted in a median difference of -4 days (0^th^ to 4^th^ quartiles: -27, -5, -4, -2 and +60 days).

The validated estimator for gestational age in Holstein fetuses based on fetometric variables consisting of a combination of head width, head length, CRL and BW was thus (Table 3):

$$Fetal\;age=32.981+0.422\;x\;head\;length+0.359\;x\;head\;width‑0.984\;x\;CRL‑0.73.41\;x\;BW+34.13\;x\;BW^{2}.$$

## Discussion

We present a validated estimator for gestational age in Holstein fetuses based on fetometric variables consisting of a combination of head width, head length, CRL and BW.

The four measurements in combination provide predictions of age of Holstein fetuses without relying on morphological characteristics, which are subjective in their nature and develop over an extended period of fetal development (S1 Table). Limited information was provided by adding the morphological characteristics data. Veterinary authorities are in great need of the presented scientifically validated fetal age estimator which we propose here, as existing estimators are based on bovine phenotypes that are often 40–50 years old, or even older, and based on data with limited background information. Furthermore, the existing estimators are based on single parameters, which make them more sensitive to measurement error or abnormal recordings in single variables.

Use of morphological data may seem appealing, but they are only useful for certain parts of the gestation period. Furthermore, due to their subjective nature, they may be subject to variation in definition and recording practices. Use of fetometric measurements are more objective and reliable, because they increase progressively over the entire gestation period, instead of being present or not. Care should be taken towards the end of gestation though, where BW, CRL and perhaps head-width exerted greater variation than earlier in gestation and because fewer data were available from the last 10% of the gestation period (Fig 1). However, head-length showed the highest R^2^ and also appeared quite close to the line. Still, the estimations at the end of the gestation period lack sufficient precision to allow definitive separation between late term fetuses in relation to be younger or older than 256 days, which is crucial in forensic cases. But the estimator can provide an expected gestation age with a calculated precision based on scientific validated data, which is greatly needed.

The study focused on Holstein cattle. Breed specific estimators are required, or the estimations should be done correcting for breed, because of the significant differences in quantitative parameters such as BW and CRL between certain breeds. Gestation age estimators have previously been developed, e.g. by Swett et al. [10] who established an estimator for Ayrshire, Guernsey, Holstein-Friesian, Jersey and mixed breed, but with averaged estimates covering 30-day intervals. The Rexroad estimator [5] provided almost similar estimates as our estimator in the validation dataset when comparing the medians, where ours was -1 day off and the Rexroad estimator was -4 days off. However, the Rexroad estimator provided greater uncertainty with 5/71 observations being more than 2 weeks from the actual gestation age based on the insemination date, whereas our estimator only included 1/274 predictions that was more than two weeks off. This prediction was -27 in our model and -29 days in the Rexroad estimator. This outlier cow also had a recorded insemination three weeks prior to the latest recorded insemination date, and the pregnancy could have been a result of the first insemination. In that case the cow’s gestation age would have fitted almost perfectly to our estimation. Although the median of our estimator was only -1 day, the predictive precision is still too poor to allow its use in forensic cases that require definitive separation between the last 10% and the preceding 90%.

The GAS was based on calculations from insemination dates recorded in the Danish Cattle Database. These dates were probably incorrect in a few cases. For example, one cow appeared with a gestation length of 25 days at slaughter according to the insemination date, but the fetometric data fitted better with a 55 days old fetus (Fig 2). As erroneous registrations always occur in breeding records in production herds, we were strict in our exclusion criteria, which may have resulted in wider prediction intervals than absolutely necessary. For example, a CRL of 65.8 cm would result in a mean prediction of a fetal age of 200 days, with a 95% prediction interval (95% PI) ranging from 185 to 215 days, whereas a HL of 171 mm would result in a mean estimate of 200 days with 95% PI of 187 to 213. If a multivariable approach is used by combining CRL = 65.8 cm, head length = 171 mm, head width = 100 mm, BW = 9.7 kg, the prediction would be 200 days, with a prediction interval of 189 to 212 days, thus adding precision.

Inclusion of all four measurements would decrease the influence of inter-observer variation in measuring the variables. These predictions can be done using the estimator (R code) in S2 Dataset. If the qualitative parameters for hair present on the coronary band in the front legs, on the dorsal part of the carpus and no hair present at the base of the tail were added to the fetometric data, the prediction was 206 days with a 95% prediction interval of 194 to 217, i.e. the precision would only slightly improve. This example provides some insight into the potential interpretation of data. The measurements are all subject to error. Care should be taken in following the instructions given in the Materials and Methods to avoid bias. Investigators should pay particular attention to the following issues: for CRL, it is important to position the fetus in lateral position with flexed neck and avoid rotation of the hind part. Ensure that the fetus is stretched and measure the longest distance between crown of forehead and the most caudal point of the thigh (Fig 1). This requires that rigor mortis is not present to allow stretching of the neck and placing the head perpendicular to the neck or that mobility of the atlanto-occipital joint is reestablished by manipulating the region. We used a perpendicular device that fixated the head in the correct position and aided straightening of the back in a uniform way, regardless of fetal size. Similar, the width of the head should be measured shortly after exsanguination of the dam and the fetal head should be protected from compression and deformation due to the softness of the only partly calcified calvarium, especially for young fetuses.

When measuring the length of the head and CRL, compression of soft tissues should be avoided. Special attention should be paid to the fontanel, which is easily compressed when measuring the length, especially during early fetal stages where the fontanel is wide and the calvarium soft. Measuring of BW is less prone to lack of precision as long as a calibrated instrument with an appropriate scale is used and BW is therefore considered the parameter with the smallest inter-observer variation. Still, measurements must be standardized, i.e. the umbilical cord must be circumcised at the interface between body wall and the umbilical cord and compression of the abdomen must be avoided to minimize esophageal reflux of the amniotic fluid normally present in the forestomachs and abomasum. If frozen specimens are examined, weighting serosanguinous fluid released during thawing is important and the head should be frozen when resting on the mandibles to avoid compression of the structures to be measured.

Determination of the gestational age in bovine fetuses is challenging due to the lack of precise reference data. Assessment of developing morphological characteristics does not have sufficient precision to be used in forensic cases and diagnostics due to the wide ranges in time of development and challenges in inter-observer variation. At best, morphological characteristics can be used to support a conclusion based on breeding data. Measurement of BW, CRL, width and length of the head are more objective, and especially BW has low inter-observer variation. Nevertheless, precision for these variables is also too low to be used in legal cases on transport and slaughter of pregnant cattle in the last 10^th^ of the gestation period. But in late term fetuses they are more useful than most morphological features as these develop mostly much earlier in gestation and, as most fetometric measurements, increased in a linear manner. Fetometric measurements are, however, highly breed dependent because of significant differences in size and body composition between breeds, e.g. Jersey versus Belgian Blue. It is therefore imperative that data on fetal parameters are investigated for all breeds or groups of comparable breeds to allow fetal age determination based on scientifically validated data, especially if such data are used in legal cases.

In conclusion, we developed a model that can be used to predict the age of Holstein fetuses with a precision of +/- 11 days if based on the fetometric measures head width and length, crown-rump length and body weight. We also identified morphological features that may be of relevant if specific periods in gestation should be investigated further, however, for the last 10^th^ of gestation, only the mentioned fetometric data were of use.

## Supporting information

S1 Fig Morphological characteristics of the bovine fetus. (PDF)

S1 Table Descriptive statistics on fetal age (days) for each stratum of each categorical variable. (DOCX)

S1 Dataset Dataset including raw data used in the study. (CSV)

S2 Dataset Estimator including dataset embedded in R code for predictions. (DOCX)
