# Supplementary material for: Fetal age assessment for Holstein cattle
Source: PLoS One. 2018 Nov 19;13(11):e0207682. doi: 10.1371/journal.pone.0207682 (PMC6242369; doi:10.1371/journal.pone.0207682)
Supplement: S1 Fig — (PDF) [file pone.0207682.s001.pdf]

Supplementary figure 1. Morphological characteristics of the bovine fetus

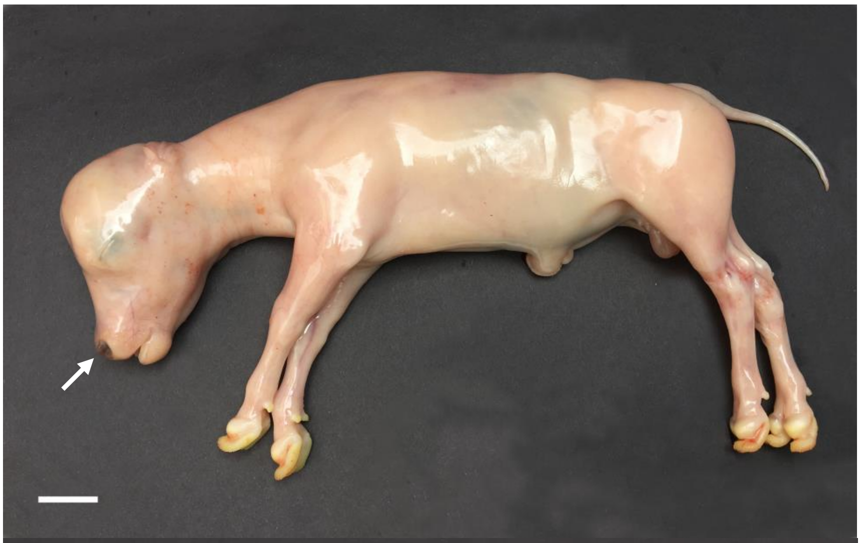

Pigmentation of the body has not yet developed and the fetus therefore lacks the dark pigmented areas of the skin that are characteristic for Holsteins. Pigmentation of the muzzle (⇨) is present. Pigmentation at this location is among the first to develop. This pattern indicates that the fetus is 88 to 93 days old. Bar = 3 cm.

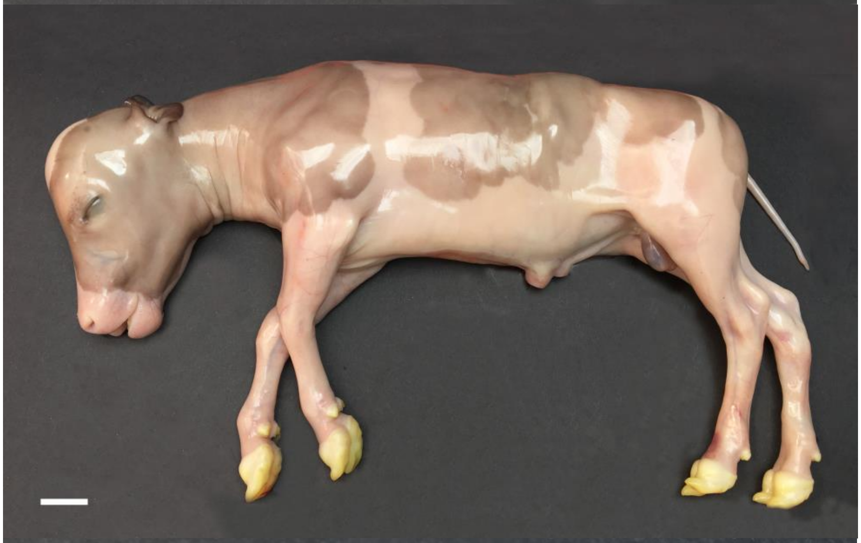

Dermal pigmentation has developed on the head and body of the fetus giving it the characteristic appearance of Holsteins. Complete pigmentation can be seen from gestation day 121. Bar = 3 cm.

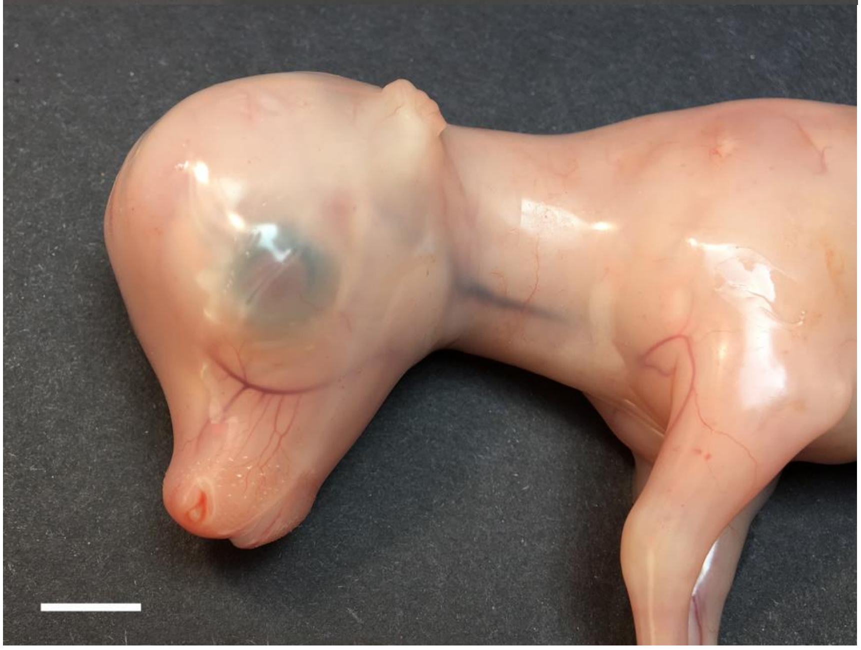

Eye lids have developed and cover the eye. The eyelids are fused. Hair follicles are present on the muzzle, but tactile hairs are not yet present. Fused eye lids and presence of hair follicles around the muzzle indicate that the fetus is between 71 and 119 day of age. Bar = 1 cm.

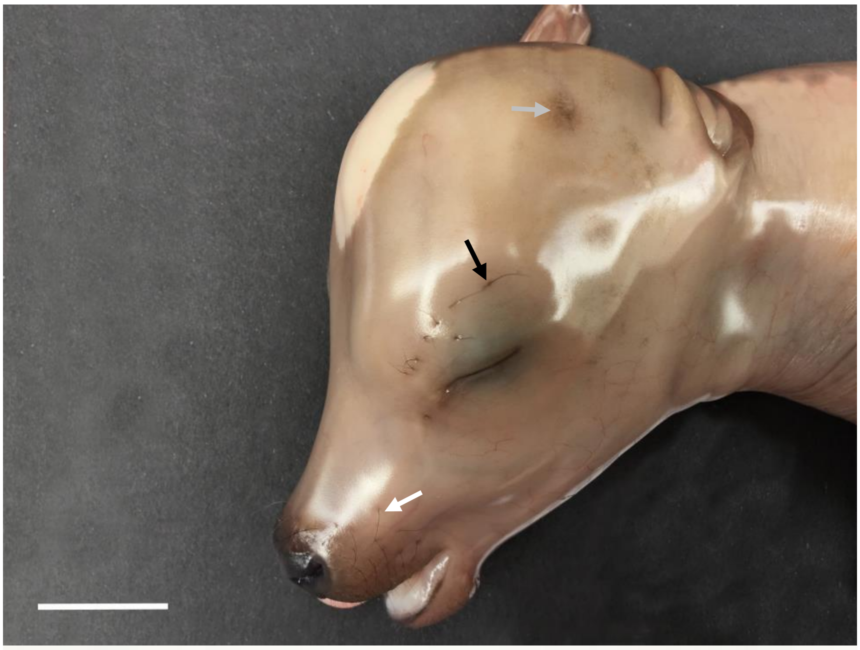

Tactile hair has developed on the muzzle (⇨) and dorsal to the eye (→), but eye lashes are not yet present. Small hairs have just developed on the horn bud (⇨). These findings indicate that the fetus is between 117 and 147 days old. Bar = 3 cm.

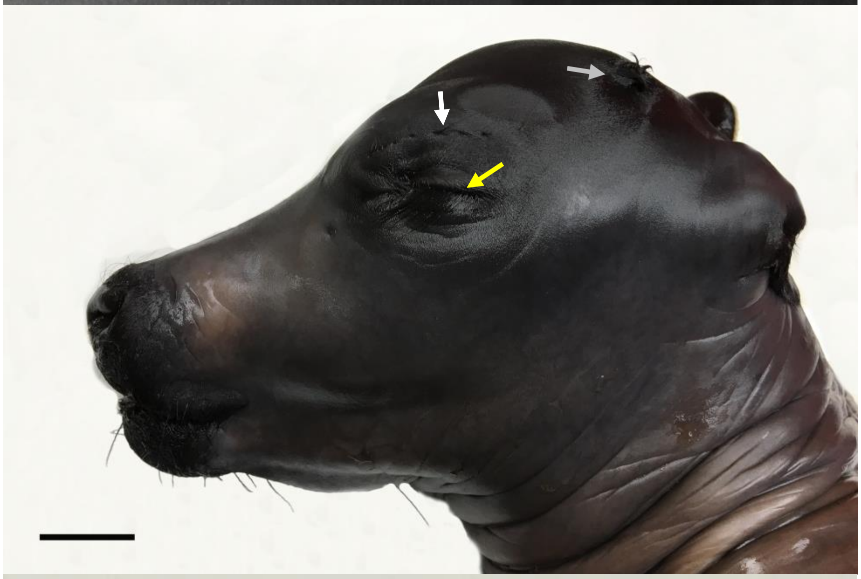

The fetus has well-developed hair on the muzzle, dorsal to the eye (⇨) and on the horn bud (⇨). Coat consisting of fine hairs are present around the eyes and well-developed eyelashes (⇨) are present. Parts of the head and the neck still lack coat. These characteristics indicate that the fetus is between 165 and 205 days old. Bar = 3 cm.

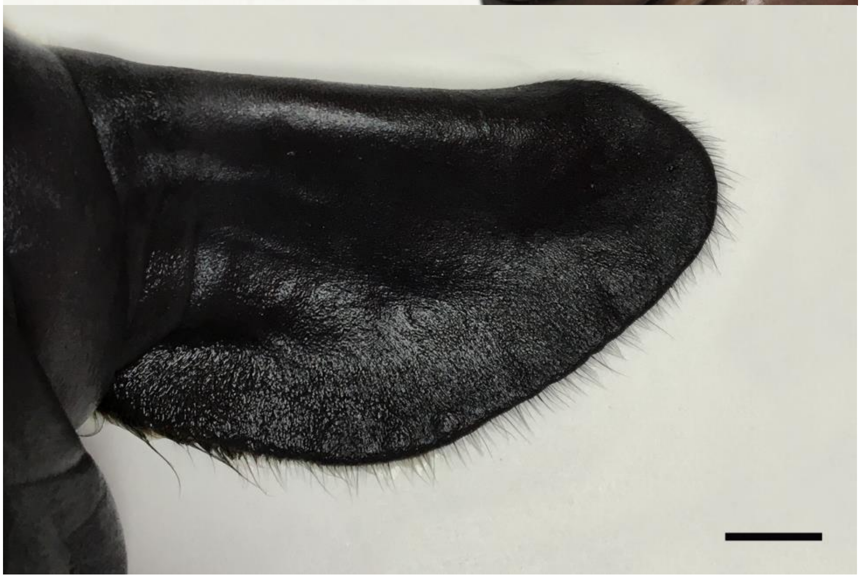

Fine hairs are present on the margin of the ear, but the skin of the ear is otherwise hairless. Bar = 1 cm.

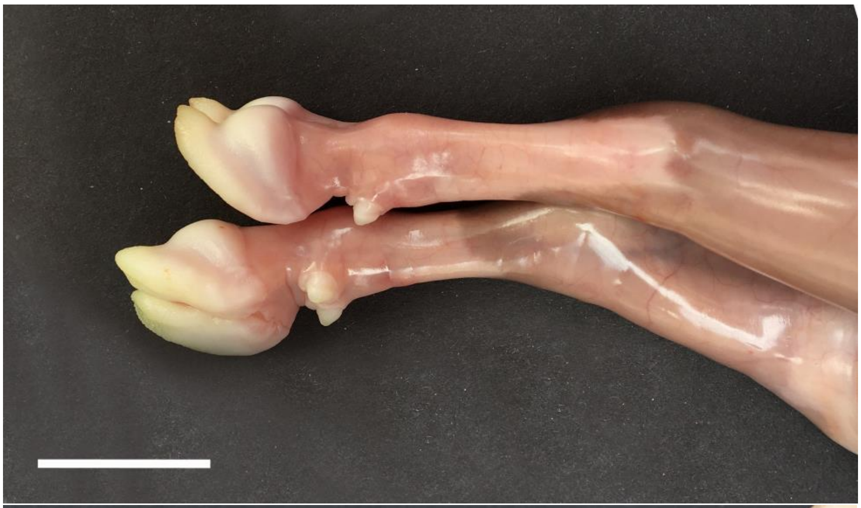

Pigmentation can be seen on the antebrachium and around the carpus on both forelimbs, but hairs are not yet developed. This indicates that the fetus is between 118 and 198 days old. Bar = 3 cm.

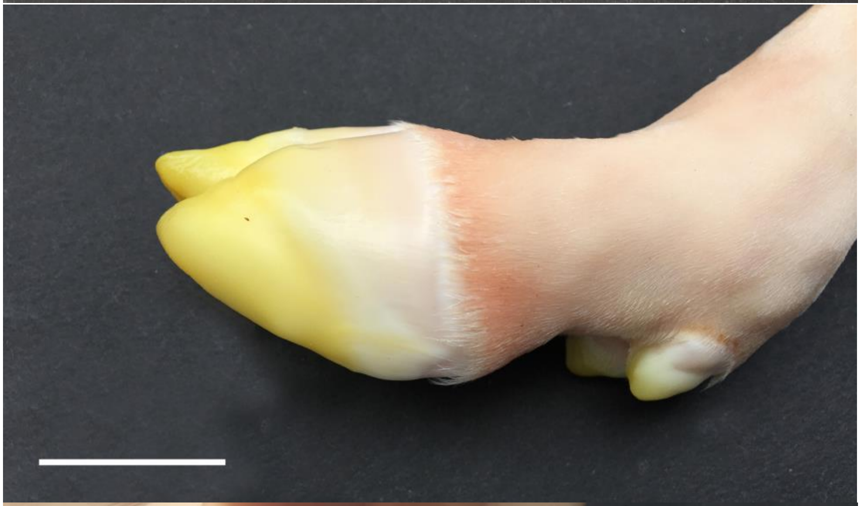

A coat consisting of fine hairs is present at the coronary band and the distal part of the limb. This indicates that the fetus is at least 202 days old. Bar = 3 cm.

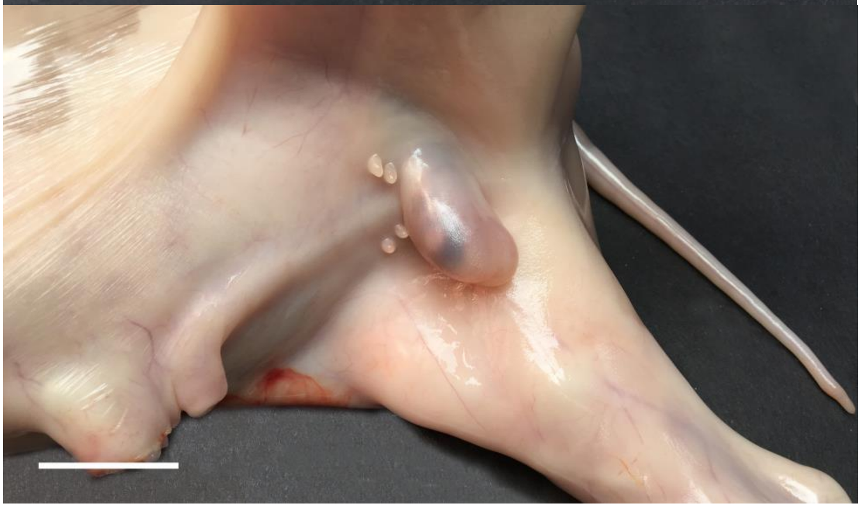

The scrotum has developed in this male fetus and one testis can be observed through the semitransparent skin of the scrotum. The testes descend around days 120-128 of gestation. Bar = 3 cm.

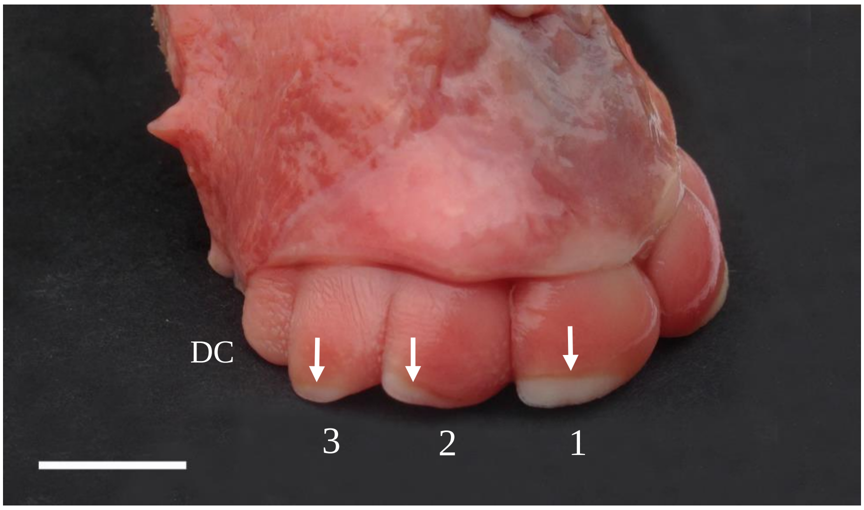

Left rostro-ventral view on the incisor teeth and dens caninum (DC) of the left mandible.The incisors are marked 1-3. All incisors of the left mandible have partially erupted though the gingiva (⇨), while DC is not erupted. Eruption of incisors occurs at the end of the gestation period from around day 224 until the neonatal period. Bar = 1 cm.
