# Supplementary material for: Fetal age assessment for Holstein cattle
Source: PLoS One. 2018 Nov 19;13(11):e0207682. doi: 10.1371/journal.pone.0207682 (PMC6242369; doi:10.1371/journal.pone.0207682)
Supplement: S1 Table — (DOCX) [file pone.0207682.s002.docx]

**S1 Table**. **Descriptive statistics on fetal age (days) for each stratum of each categorical variable**

| **Variable** | **Stratum** | **N** | **Min.** | **Percentiles** | | | | | **Max** |
| --- | --- | --- | --- | --- | --- | --- | --- | --- | --- |
|  |  |  |  | **2.5** | **5** | **50** | **95** | **97.5** |  |
| Gender differentiation | No | 24 | 37 | 37 | 37 | 46 | 60 | 60 | 61 |
|  | Yes | 250 | 25 | 61 | 71 | 146 | 222 | 237 | 274 |
| Genital tubercle | Yes | 45 | 25 | 37 | 37 | 54 | 86 | 93 | 95 |
|  | No | 229 | 71 | 78 | 81 | 151 | 223 | 238 | 274 |
| Testicular descent | None | 35 | 25 | 35 | 61 | 91 | 121 | 128 | 130 |
|  | Unilateral | 1 | 121 | 121 | 121 | 121 | 121 | 121 | 121 |
|  | Full | 98 | 111 | 120 | 122 | 170 | 235 | 245 | 259 |
|  | NA | 140 | 37 | 43 | 45 | 121 | 221 | 228 | 274 |
| Incisor 1 (R) | Not present | 247 | 25 | 44 | 46 | 128 | 193 | 198 | 214 |
|  | Not erupted | 17 | 201 | 203 | 205 | 215 | 229 | 232 | 235 |
|  | Erupted | 10 | 223 | 224 | 225 | 244 | 270 | 272 | 274 |
| Incisor 2 (R) | Not present | 262 | 25 | 44 | 46 | 133 | 207 | 214 | 235 |
|  | Not erupted | 6 | 219 | 219 | 220 | 225 | 238 | 239 | 239 |
|  | Erupted | 6 | 237 | 238 | 240 | 256 | 272 | 273 | 274 |
| Incisor 3 (R) | Not present | 260 | 25 | 44 | 46 | 133 | 206 | 214 | 228 |
|  | Not erupted | 11 | 217 | 218 | 218 | 235 | 262 | 263 | 264 |
|  | Erupted | 3 | 249 | 249 | 249 | 252 | 272 | 273 | 274 |
| Dens caninum (R) | Not erupted | 1 | 274 | 274 | 274 | 274 | 274 | 274 | 274 |
|  | Not present | 273 | 25 | 44 | 47 | 137 | 220 | 235 | 264 |
| Incisor 1 (L) | Not present | 248 | 25 | 44 | 46 | 128 | 194 | 198 | 214 |
|  | Not erupted | 15 | 201 | 203 | 205 | 215 | 226 | 230 | 235 |
|  | Erupted | 11 | 223 | 224 | 225 | 239 | 269 | 272 | 274 |
| Incisor 2 (L) | Not present | 262 | 25 | 44 | 46 | 133 | 207 | 214 | 235 |
|  | Not erupted | 8 | 219 | 220 | 220 | 232 | 252 | 256 | 259 |
|  | Erupted | 4 | 249 | 249 | 249 | 258 | 272 | 273 | 274 |
| Incisor 3 (L) | Not present | 260 | 25 | 44 | 46 | 133 | 206 | 214 | 228 |
|  | Not erupted | 10 | 217 | 217 | 218 | 231 | 253 | 258 | 264 |
|  | Erupted | 4 | 249 | 249 | 249 | 256 | 272 | 273 | 274 |
| Dens caninum (L) | Not erupted | 1 | 274 | 274 | 274 | 274 | 274 | 274 | 274 |
|  | Not present | 273 | 25 | 44 | 47 | 137 | 220 | 235 | 264 |
| Hair, coronay band (front) | No | 210 | 25 | 42 | 45 | 120 | 173 | 175 | 183 |
|  | Yes | 64 | 176 | 177 | 177 | 200 | 252 | 261 | 274 |
| Hair, coronay band (back) | No | 213 | 25 | 43 | 45 | 121 | 175 | 177 | 183 |
|  | Yes | 61 | 176 | 178 | 180 | 201 | 252 | 262 | 274 |
| Hair, carpus | No | 231 | 25 | 44 | 46 | 124 | 184 | 188 | 221 |
|  | Yes | 43 | 184 | 185 | 188 | 212 | 258 | 264 | 274 |
| Hair, tarsus | No | 247 | 25 | 44 | 46 | 128 | 193 | 198 | 221 |
|  | Yes | 27 | 198 | 202 | 205 | 221 | 262 | 268 | 274 |
| Hair, ear – inside | No | 230 | 25 | 43 | 45 | 124 | 185 | 188 | 196 |
|  | Yes | 44 | 173 | 184 | 185 | 213 | 258 | 264 | 274 |
| Hair, ear – base | No | 164 | 25 | 42 | 44 | 106 | 149 | 152 | 156 |
|  | Yes | 110 | 151 | 159 | 159 | 184 | 238 | 254 | 274 |
| Hair, eyelids | No | 205 | 25 | 42 | 45 | 119 | 173 | 176 | 188 |
|  | Yes | 69 | 151 | 165 | 174 | 197 | 251 | 260 | 274 |
| Hair, tail | No | 186 | 25 | 42 | 45 | 115 | 161 | 163 | 176 |
|  | Yes | 88 | 164 | 166 | 169 | 190 | 245 | 258 | 274 |
| Hair, dorsum | No | 254 | 25 | 44 | 46 | 130 | 198 | 206 | 217 |
|  | Yes | 20 | 214 | 214 | 214 | 228 | 264 | 269 | 274 |
| Hair, corpus | No | 252 | 25 | 44 | 46 | 130 | 196 | 203 | 215 |
|  | Yes | 22 | 211 | 213 | 214 | 225 | 264 | 269 | 274 |
| Hair, horn buds | No | 182 | 25 | 42 | 45 | 114 | 162 | 168 | 193 |
|  | Yes | 92 | 150 | 155 | 161 | 188 | 244 | 257 | 274 |
| Coat | No | 164 | 25 | 42 | 44 | 106 | 149 | 152 | 219 |
|  | Yes | 110 | 150 | 154 | 159 | 184 | 238 | 254 | 274 |
| Complete coat | No | 258 | 25 | 44 | 46 | 132 | 204 | 213 | 235 |
|  | Yes | 16 | 196 | 205 | 213 | 232 | 266 | 270 | 274 |
| Hair, rear end of the fetus | No | 260 | 25 | 44 | 46 | 133 | 206 | 214 | 235 |
|  | Yes | 14 | 221 | 221 | 221 | 237 | 268 | 271 | 274 |
| Pigmentation, muzzle | No | 54 | 25 | 37 | 38 | 58 | 85 | 108 | 133 |
|  | Yes | 220 | 79 | 86 | 91 | 155 | 223 | 238 | 274 |
| Pigmentation, eyelids | No | 67 | 25 | 37 | 40 | 70 | 92 | 95 | 100 |
|  | Yes | 207 | 87 | 99 | 103 | 160 | 226 | 239 | 274 |
| Pigmentation, lips | No | 58 | 25 | 37 | 39 | 60 | 87 | 93 | 119 |
|  | Yes | 216 | 79 | 88 | 95 | 157 | 224 | 238 | 274 |
| Pigmentation, ears | No | 68 | 25 | 37 | 40 | 70 | 97 | 100 | 104 |
|  | Yes | 206 | 85 | 98 | 103 | 160 | 226 | 239 | 274 |
| Pigmentation, legs | No | 105 | 25 | 38 | 42 | 81 | 121 | 127 | 165 |
|  | Yes | 169 | 106 | 118 | 121 | 169 | 232 | 247 | 274 |
| Pigmentation, neck | No | 97 | 25 | 38 | 42 | 79 | 118 | 121 | 164 |
|  | Yes | 177 | 103 | 115 | 118 | 166 | 229 | 245 | 274 |
| Pigmentation, tail | No | 106 | 25 | 38 | 42 | 82 | 121 | 127 | 164 |
|  | Yes | 168 | 106 | 118 | 122 | 169 | 233 | 247 | 274 |
| Pigmentation, dorsum | No | 106 | 25 | 38 | 42 | 82 | 121 | 127 | 164 |
|  | Yes | 168 | 106 | 118 | 122 | 169 | 233 | 247 | 274 |
| Pigmentation, complete | No | 109 | 25 | 38 | 43 | 84 | 121 | 126 | 164 |
|  | Yes | 165 | 106 | 121 | 122 | 170 | 234 | 248 | 274 |
| Tactile hair, muzzle | Not visible | 34 | 25 | 35 | 37 | 48 | 65 | 70 | 82 |
|  | Hair follicle | 63 | 70 | 71 | 71 | 93 | 118 | 119 | 123 |
|  | Visible | 177 | 106 | 117 | 120 | 166 | 229 | 245 | 274 |
| Tactile hair, eyebrow | Not visible | 45 | 25 | 37 | 37 | 54 | 79 | 81 | 82 |
|  | Hair follicle | 52 | 70 | 75 | 77 | 98 | 118 | 120 | 123 |
|  | Visible | 177 | 106 | 117 | 120 | 166 | 229 | 245 | 274 |
| Tactile hair, eyelash | Not visible | 135 | 25 | 40 | 44 | 95 | 144 | 147 | 155 |
|  | Hair follicle | 24 | 115 | 117 | 118 | 134 | 158 | 163 | 166 |
|  | Visible | 115 | 138 | 149 | 153 | 181 | 238 | 253 | 274 |
| Eyelids | Closed | 235 | 25 | 44 | 46 | 127 | 188 | 193 | 207 |
|  | Open | 39 | 137 | 181 | 185 | 214 | 260 | 264 | 274 |
| Tongue papillae | None | 36 | 25 | 36 | 37 | 49 | 71 | 73 | 82 |
|  | Furthest back | 29 | 61 | 68 | 71 | 80 | 107 | 116 | 122 |
|  | Large front | 37 | 80 | 85 | 87 | 106 | 124 | 128 | 135 |
|  | Whole tongue | 172 | 106 | 115 | 120 | 169 | 231 | 246 | 274 |
| Eyelids | Yes | 253 | 25 | 59 | 71 | 146 | 221 | 237 | 274 |
|  | No | 21 | 37 | 37 | 37 | 45 | 54 | 54 | 55 |

R: right; L: left
