# Supplementary material for: Fetal age assessment for Holstein cattle
Source: PLoS One. 2018 Nov 19;13(11):e0207682. doi: 10.1371/journal.pone.0207682 (PMC6242369; doi:10.1371/journal.pone.0207682)
Supplement: S2 Dataset — (DOCX) [file pone.0207682.s004.docx]

S2 Dataset. Estimator including dataset embedded in R code for predictions

# Fetal age estimator -----------------------------------------------------

# Instructions ------------------------------------------------------------

#This estimator is based on the study presented in Hessel C, Agerholm JS, Nielsen S, Fetal age assessment for Holstein cattle. PloS One.

#To use this estimator to estimate the age of a specific Holstein calf with one of more of the variables

# head width (in mm),

# head lenght (in mm),

# crown-rump lenght (in cm)

# weight (kg)

# you must do the following:

# 1) Load the data

# 2) Run the models

# 3) Estimate age of a specific calf

# Loading the data from the paper --------------------------------------------------------

# Data frame including quantitative variables from Hessel C, Agerholm JS, Nielsen S, Fetal age assessment for Holstein cattle. PloS One.

#Highlight data from here to where it says ""end of highlighting data" and press "Ctrl+Enter" (in R studio)

data <- structure(list(age_days =

c(197, 274, 196, 259, 249, 221, 221, 177, 82, 193, 142, 227, 169, 143, 177, 196, 110, 181, 175, 180, 111, 211, 121, 88, 118, 130, 181, 118, 211, 134, 151, 146, 133,78, 150, 212, 173, 120, 153, 114, 198, 77, 173, 177, 161, 163, 156, 111, 123, 228, 86, 111, 94, 161, 98, 155, 129, 166, 185, 204, 71, 103, 116, 100, 75, 71, 76, 109, 105, 183, 122, 118, 201, 98, 81, 86, 180, 63, 133, 137, 55, 175, 152, 162, 60, 173, 91, 101, 144, 113, 81, 137, 184, 191, 133, 89, 147, 175, 78, 68, 61, 87, 214, 80, 160, 93, 172, 72, 96, 100, 167, 215, 70, 161, 59, 129, 80, 159, 127, 146, 54, 113, 145, 85, 61, 147, 103, 88, 84, 56, 95, 128, 78, 152, 214, 201, 106, 53, 121, 49, 79, 128, 88, 149, 109, 127, 140, 122, 128, 115, 74, 185, 79, 117, 164, 115, 175, 54, 158, 172, 127, 58, 173, 104, 147, 125, 100,99, 147, 187, 146, 189, 135, 146, 101, 51, 58, 122, 206, 160, 48, 71, 124, 44, 47, 237, 166, 46, 138, 77, 155, 159, 128, 92,47, 140, 133, 176, 179, 139, 162, 122, 173, 222, 45, 132, 119, 45, 118, 160, 115, 165, 121, 49, 46, 121, 169, 194, 187, 173, 169, 188, 164, 131, 188, 159, 161, 42, 188, 45, 235, 121, 44, 135, 142, 170, 169, 42, 190, 184, 178, 119, 202, 206, 79, 194,176, 37, 188, 193, 185, 214, 44, 37, 118, 198, 150, 252, 176, 217, 237, 175, 162, 39, 207, 191, 151, 219, 239, 215, 106, 25,223, 264),

head_length_mm = c(144.42, 239, 154, 220.1, 215, 183, 189, 142.19, 36.8, 153.1, 99.84, 199.1, 131.7, 103.9, 139.6, 163.2, 71.3, 144.3, 140, 142.7, 71.75, 176, 79.8, 45.96, 76.03, 90.78, 145.6, 75.9, 176, 95.53, 113.9, 117, 93.3, 36.98, 107.2, 183, 135.7, 81.8, 116.6, 74.93, 162, 37.86, 141.9, 145.38, 130, 127.7, 120, 74.8, 87.26, 201, 42.89, 73.03, 52.54, 122.86, 58.5,119, 93.9, 132.6, 147, 175, 31.4, 63.54, 77.13, 58.66, 36.05, 34.34, 38.1, 68.74, 64.8, 153, 83.9, 80.02, 168.1, 57.98, 41.36,43.81, 150, 26.65, 99.65, 99.8, 18.32, 143, 114, 131, 23.43, 132.2, 53.1, 60.83, 108.23, 79.74, 44.4, 101.6, 149, 170, 97.5, 49.23, 110.69, 136.7, 40.31, 30.88, 25.55, 47.73, 179, 40.02, 126.38, 52.96, 132.5, 36.8, 54.1, 57.1, 137.8, 189, 29.9, 128.29, 22.61, 95.8, 40, 128.42, 93.31, 116.8, 17.58, 78.91, 108.7, 47.09, 24.85, 112.4, 64.15, 49.51, 49, 21.66, 57.85, 90.62, 42.39, 117.7,188, 173.1, 68.02, 17.11, 82.22, 12.7, 40.74, 94.31, 47.73, 118.5, 65.5, 88.73, 105.84, 89.44, 92.1, 76.7, 36.39, 159.2, 40.33, 82.1, 140, 80.43, 146.6, 18.51, 121.4, 156, 92.29, 23.2, 145.2, 65.31, 114.3, 91.04, 61.27, 61.82, 114.5, 156, 117.5, 158, 100.67, 109.9, 61.65, 19.01, 22.79, 86.88, 161.5, 127.61, 14.26, 34.64, 89.59, 9.14, 11.96, 198, 141.2, 11.9, 108.1, 40.48, 121, 124.8, 92.64, 54.99, 14.48, 112.2, 101.29, 149, 160, 101.28, 132.18, 91.4, 152, 188.6, 12.57, 93.2, 86.51, 10, 84.84, 130.8, 81.52, 137, 90.05, 15.6, 13.1, 88.8, 134, 166.5, 170, 147.9, 137.7, 169.2, 142, 110.2, 155.1, 144, 140, 8.84, 168, 13.5, 212, 93.98, 14, 103.06, 111, 136.3, 157, 11.75, 169, 156.3, 148, 98.2, 184.2, 186, 45.08, 173, 151, 7.3, 155, 178, 166, 178, 14, 5, 79.1, 173, 122.2, 232, 158, 198, 219, 152.2, 124.8, 15, 215, 171.2, 141, 192, 223, 193, 95.83, 23.83, 194.3, 233.1),

head_width_mm = c(84.61, 128, 83.79, 121, 118, 100.6, 97, 74.52, 24.28, 85.39, 62.37, 105.1, 77.28, 63.05, 79.96, 93.01, 43.6, 83.45, 80.1, 82.3, 45.16, 101, 50.67, 31.24, 50.4, 56.81, 83.28, 49.15, 103, 60.11, 69.31, 66.2, 59.98, 25.54, 70.3, 102.41, 83.37, 50.2, 71.52, 48.45, 93.6, 24.96, 82.69, 84.93, 74.55, 77.56, 73.7, 47, 52.99, 110, 31.98, 46.99, 36.38, 75.78, 38.1, 71, 57.9, 78.7, 90.15, 98.31, 21.03, 42.4, 50.63, 40.74, 24.58, 22.08, 25.71, 46.94, 43.92,88.45, 54.59, 52.05, 95.9, 39.65, 29.03, 32.45, 82.72, 16.03, 59.26, 63.19, 10.67, 82.69, 71.2, 75.6, 13.8, 83.2, 35, 40.87, 68.21, 46.57, 27.44, 62.66, 87.26, 88.1, 61.19, 33.43, 67.83, 85.11, 27.38, 19.91, 15.14, 33.12, 106, 29.79, 77.1, 37.86, 83.4, 22.43, 41, 43.5, 78.8, 104.1, 22.32, 77.8, 14.2, 59.09, 29.11, 74.47, 56.23, 67.61, 11.24, 48.47, 68.8, 31.99, 15.8, 69.92, 45.15, 33.87, 30.92, 12.43, 39.11, 59.22, 27.73, 73.08, 103, 98.1, 46.92, 10.74, 56.15, 8.17, 29.18, 58.59, 36, 72, 48.97, 59.05, 65.81, 55.28, 58.9, 51.59, 25.59, 89.15, 28.68, 52.19, 80.2, 50.54, 83.14, 12.25, 77.2, 80.1, 60.04, 15.1, 82.7, 46.01, 68.37, 57.37, 43.58, 42.54, 69.2, 93, 68.3, 93.3, 63.47, 70, 44.4, 10.05, 14.93, 56.38, 106.6, 76.09, 8.52, 23.7, 57.28, 6.8, 8.58, 124, 80.49, 7.7, 66.06, 28.75, 74.53, 77.1, 59.87, 40.1, 8.7, 66.23, 62.97, 86.8, 88.19, 67.59, 79.12, 56.4, 85.45, 114.2, 7.48, 64.3, 55.12, 8.45, 55.79, 78.67, 54.98, 80.68, 55.64, 10.52, 8.2, 58.23, 79.88, 96.11, 90.57, 84.1, 84.7, 92.1, 78.38, 60.14, 96.56, 75.6, 76.14, 6.9, 94.51, 8.59, 120.8, 56.65, 7.1, 68.85, 69.45, 86.9, 82.1, 7.44, 95.5, 93.67, 93.11, 55.3, 103, 103.2, 33.24, 100.22, 87, 3.8, 98.7, 96.76, 93.48, 113.7, 10, 3, 61.92, 97.31, 76.92, 132, 90.38, 112, 119, 90.8, 89.5, 8.11, 106, 111.1, 81.52, 122.2, 137, 124, 64.5, 16.18, 152, 120.1),

weight_kg = c(6.45, 35.5, 10.25, 27.15, 29.1, 16.55, 19.05, 6.35, 0.046, 11.15, 1.771, 18.55, 4.85, 1.86, 6.35, 11.55, 0.474, 7.45, 5.45, 8.35, 0.525, 12.45, 0.853, 0.152, 0.6, 1.645, 7.25, 0.729, 18.35, 1.516, 3.75, 1.997, 1.441, 0.066, 3.05, 13.55, 7.15, 0.92, 3.069, 0.252, 12.65, 0.06, 6.55, 6.95, 4.35, 4.25, 3.45, 0.644, 1.012, 19.75, 0.136, 0.611, 0.215, 3.45, 0.283, 3.85, 1.36, 5.35, 8.75, 12.45, 0.039, 0.38, 0.764, 0.373, 0.06, 0.041, 0.069, 0.585, 0.477, 7.55, 0.983, 0.833, 13.65, 0.311, 0.096, 0.139, 7.35, 0.019, 1.501, 1.79, 0.007, 6.65, 3.25, 4.35, 0.012, 7.55, 0.211, 0.325, 2.453, 0.654, 0.102, 1.813, 7.95, 10.55, 1.429, 0.173, 2.622, 6.15, 0.079, 0.029, 0.016, 0.174, 16.85, 0.088, 4.55, 0.209, 6.05, 0.047, 0.278, 0.387, 5.25, 17.05, 0.034, 3.65, 0.011, 1.496, 0.089, 4.75, 1.177, 3.55, 0.006, 0.612, 3.05, 0.139, 0.027, 2.374, 0.402, 0.165, 0.123, 0.103, 0.259, 1.163, 0.084, 3.95, 17.25, 12.55, 0.486, 0.006, 1.147, 0.003, 0.088, 1.174, 0.182, 3.288, 0.601, 1.189, 2.367, 1.004, 1.327, 0.815, 0.057, 9.15, 0.104, 0.816, 5.15, 0.89, 6.85, 0.008, 3.95, 6.15, 1.46, 0.014, 6.35, 0.395, 2.95, 1.378, 0.397, 0.364, 2.95, 11.85, 3.05, 9.95, 1.67, 2.75, 0.411, 0.006, 0.015, 1.141, 14.85, 3.85, 0.003, 0.053, 1.227, 0.001, 0.003, 28.95, 5.35, 0.003, 2.126, 0.094, 5.95, 4.25, 1.521, 0.215, 0.003, 3.35, 1.449, 6.95, 8.35, 2.011, 4.65, 1.137, 6.15, 19.75, 0.002, 1.75, 0.984, 0.002, 1.032, 3.85, 0.651, 4.85, 1.083, 0.004, 0.003, 1.053, 4.85, 9.95, 9.35, 7.05, 5.75, 9.85, 4.45, 1.691, 10.15, 3.65, 4.95, 0.003, 9.95, 0.003, 20.35, 1.004, 0.002, 2.002, 2.456, 6.35, 5.95, 0.002, 11.55, 9.65, 7, 0.965, 14.55, 15.75, 0.092, 11.55, 7.55, 0.001, 10.35, 10.75, 9, 18.75, 0.002, 0.003, 0.746, 13.05, 3.55, 36, 7.15, 21.75, 28.75, 7.85, 4.45, 0.003, 19.05, 10.15, 6.55, 20.75, 25.25, 19.55, 1.197, 0.017, 21.5, 41.6),

crl_cm = c(53.3, 86.8, 61.8, 89.3, 83, 75.4, 77.7, 55.4, 10.4, 62.4, 35.4, 77.2, 46.1, 36.3, 54.8, 60, 22.9, 56.2, 52.7, 56.7, 23.6, 70.9, 28.4, 15.5, 26.4, 31.2, 56.8, 27.2, 68.3, 33, 40.9, 37.4, 31.6, 12, 41.1, 68.8, 48.9, 28.7, 40.7, 24.3, 66, 11.8, 50.6, 52, 46, 45.7, 43.4, 23.5, 30.3, 79.4, 15.2, 24.2, 17.7, 46, 19.4, 44.4, 31, 47.2, 57.6, 66.9, 10, 20.4, 26.8, 20.8, 11.3, 9.8, 10.4, 23.8, 23.1, 55.6, 29.2, 26.4, 69.3, 19.8, 13.6, 14.9, 59.4, 7.47, 33.9, 35.2, 4.6, 54.2, 43, 47.8, 6, 53.2, 17.2, 21.2, 37.4, 26.6, 13.7, 35.7, 59.6, 63.8, 32.4, 16.3, 41.6, 54.6, 12.7, 9.3, 7.5, 16.35, 74.1, 13, 45.9, 17.8, 53.2, 10.6, 18.2, 19.8, 50, 74.9, 9.9, 45.5, 6.2, 31.9, 13.6, 46.9, 32.2, 40.2, 4.1, 26, 39.4, 14.8, 6.98, 39.8, 21.7, 17.1, 15.2, 5.7, 18.6, 32, 12.7, 43.2, 77, 67.3, 22.3, 4.5, 29.2, 3, 13.3, 32.3, 15.7, 39.4, 24.7, 31.3, 37.9, 29.5, 32.5, 26.7, 11.2, 59.3, 14.3, 27.3, 44.6, 27.2, 55, 5.1, 46.8, 54.5, 30.4, 6.97, 54.6, 23, 44.1, 31.4, 21, 20.7, 43.2, 60.1, 42, 61, 35.6, 41.7, 21.7, 4, 6.9, 29.5, 68.5, 49.8, 3.3, 11.2, 32, 2.4, 3.1, 83.6, 50.3, 2.8, 36.3, 13.1, 46.8, 49.4, 34.5, 17.6, 3.1, 38.8, 35.2, 54.8, 54, 37.9, 48.4, 30.6, 53, 78.8, 2.6, 36.2, 29.8, 2.7, 28.3, 48.8, 25.3, 51.1, 31, 3.6, 2.9, 28.2, 58.8, 65.5, 61.1, 55.7, 52.4, 61.5, 50.7, 36.6, 62, 47.6, 51.8, 2.4, 60.8, 3.1, 83, 30, 2.4, 33.4, 40, 52.9, 52, 2.6, 63.2, 59.6, 53.5, 28.7, 66.7, 71.6, 13.4, 63.7, 60.2, 2, 61, 64.3, 60.1, 74.2, 3.4, 5.4, 27.7, 75.4, 44.2, 93.5, 56.7, 78.4, 93.2, 57, 47.7, 3.2, 76.8, 61.2, 50.3, 86.2, 86.9, 78.5, 33.3, 7.5, 84.3, 101.3)),

.Names = c("age_days", "head_length_mm", "head_width_mm", "weight_kg", "crl_cm"), row.names = c(NA, -274L), class = c("tbl_df", "tbl", "data.frame"))

#end of highlighting data

# 2) Create the 5 estimators ----------------------------------------------

# Highlight the 5 lines below and press "Ctrl+Enter"

est.crl <- lm(age_days ~ crl_cm, data=data)

est.hw <- lm(age_days ~ head_width_mm, data=data)

est.hl <- lm(age_days ~ head_length_mm, data=data)

est.weight <- lm(age_days ~ poly(weight_kg,4), data=data)

est.4var <- lm(age_days ~ crl_cm + head_width_mm + head_length_mm + poly(weight_kg,4), data=data)

# 3) Do predictions by replacing the specific values in examples --------

# Replace the head width, head length, crown-rump length and/or weight with the data from your calf

# Or just use the data already there.

# Highlight the line and press "Ctrl+Enter" (in R studio)

# 3a) Crown-rump length only ----------------------------------------------

(pred.crl <- round(predict(est.crl,newdata=data.frame(crl_cm=65.8),interval='prediction'),0))

# 3b) Head width only -----------------------------------------------------

(pred.hw <- round(predict(est.hw,newdata=data.frame(head_width_mm=100),interval='prediction'),0))

# 3c) Head length only ----------------------------------------------------

(pred.hl <- round(predict(est.hl,newdata=data.frame(head_length_mm=171),interval='prediction'),0))

# 3d) Weight only ---------------------------------------------------------

(pred.weight <- round(predict(est.weight,newdata=data.frame(weight_kg=9.7),interval='prediction'),0))

# 3e) All four variables --------------------------------------------------

(pred.4var <- round(predict(est.4var,newdata=data.frame(crl_cm=65.8,head_width_mm=100,head_length_mm=171,weight_kg=9.7),interval='prediction'),0))
